# Narrow‐band imaging with magnification for the diagnosis of colorectal adenoma in a patient with Cronkhite‐Canada syndrome

**DOI:** 10.1002/deo2.257

**Published:** 2023-06-15

**Authors:** Hitoshi Fukase, Munenori Honda, Hideaki Miyamoto, Masatoshi Nakashima, Ryosuke Gushima, Hideaki Naoe, Rin Yamada, Yoshihiro Komohara, Yasuhito Tanaka

**Affiliations:** ^1^ Department of Gastroenterology and Hepatology Kumamoto University Hospital Kumamoto Japan; ^2^ Department of Cell Pathology Graduate School of Medical Sciences Kumamoto University Kumamoto Japan

**Keywords:** adenoma, colonoscopy, Cronkhite–Canada syndrome, hamartoma, narrow‐band imaging

## Abstract

Cronkhite‐Canada syndrome (CCS) is a rare disease characterized by gastrointestinal polyposis, skin pigmentation, alopecia, and abnormal nailfolds. Although colorectal cancer has been reported in patients with CCS, reports are limited regarding the effectiveness of the usage of image‐enhanced endoscopy in CCS lesions. Here, we report a case of CCS in which narrow‐band imaging (NBI) magnifying endoscopy was applied to detect an adenomatous component in multiple hamartomatous polyps. A 79‐year‐old female complained of taste disorder, anorexia, and weight loss over several months. Endoscopic examination revealed multiple reddened polyps in the stomach and colon, leading to a diagnosis of CCS. Narrow‐band imaging magnification showed sparse and dilated round pits on the CCS polyps. Furthermore, 12 out of the numerous colorectal CCS polyps had a coexisting light reddish elevated component with a regular distribution of microvessels and a regular reticular pattern. This pattern satisfied the criteria for Type 2A of the Japan Narrow‐band‐imaging Expert Team classification, indicating adenoma. After resection, these twelve polyps were subject to pathological analysis, which confirmed they were all hamartomatous polyps with low‐grade adenoma on the superficial layer. Immunohistochemical analysis revealed a significant increase in the Ki‐67 index and p53 staining only in the adenomatous lesions. We conclude that narrow‐band imaging magnifying endoscopy would be useful in differentiating adenoma from CCS‐related polyps, which thereby facilitates early detection and treatment of precancerous lesions.

## INTRODUCTION

Cronkhite‐Canada syndrome (CCS) is mainly characterized by gastrointestinal polyposis, skin pigmentation, alopecia, and abnormal nailfolds.^1^ Although the gastrointestinal lesions associated with CCS are generally classified as juvenile‐like or hamartomatous polyposis, colorectal adenoma, and carcinoma occasionally coexist with CCS lesions. However, there are limited reports on the effectiveness of the use of image‐enhanced endoscopy in the observation of CSS lesions.

In this context, we report here a case of CCS in which magnifying endoscopy with narrow‐band imaging (NBI) enabled discrimination of an adenomatous component among multiple hamartomatous polyps. Histological analysis revealed some adenomatous lesions accompanied by hamartomatous polyps, implying several possibilities of tumorigenic pathways in this CSS patient.

## CASE REPORT

A 79‐year‐old female was referred to our hospital, complaining of taste disorder, anorexia, and loss of 10 kg bodyweight over several months. She had no family history of gastrointestinal polyposis, and no abnormal findings were reported in the previous colonoscopy that was performed 3 years before this latest referral. Physical examination revealed nail dystrophy as well as hyperpigmentation on her face and extremities. The patient did not have alopecia. Initial laboratory examinations indicated hypoproteinemia (total protein 5.3 g/dl and albumin 3.0 g/dl), but there was no elevation of tumor markers such as CEA or CA19‐9.

**FIGURE 1:**
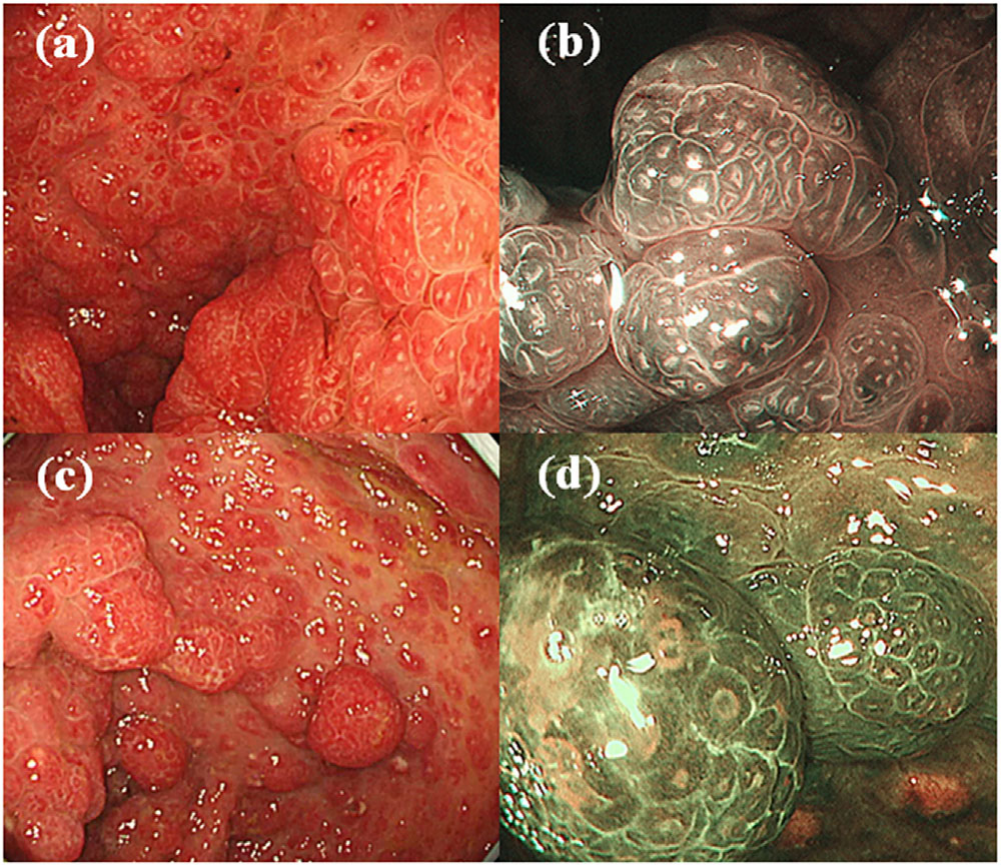
Esophagogastroduodenoscopy and colonoscopy findings. Esophagogastroduodenoscopy revealed a dense strawberry‐like polyposis in the stomach (a, b). Colonoscopy showed a dense strawberry‐like polyposis (c, d). NBI magnification showed sparse and dilated round pits.

**FIGURE 2:**
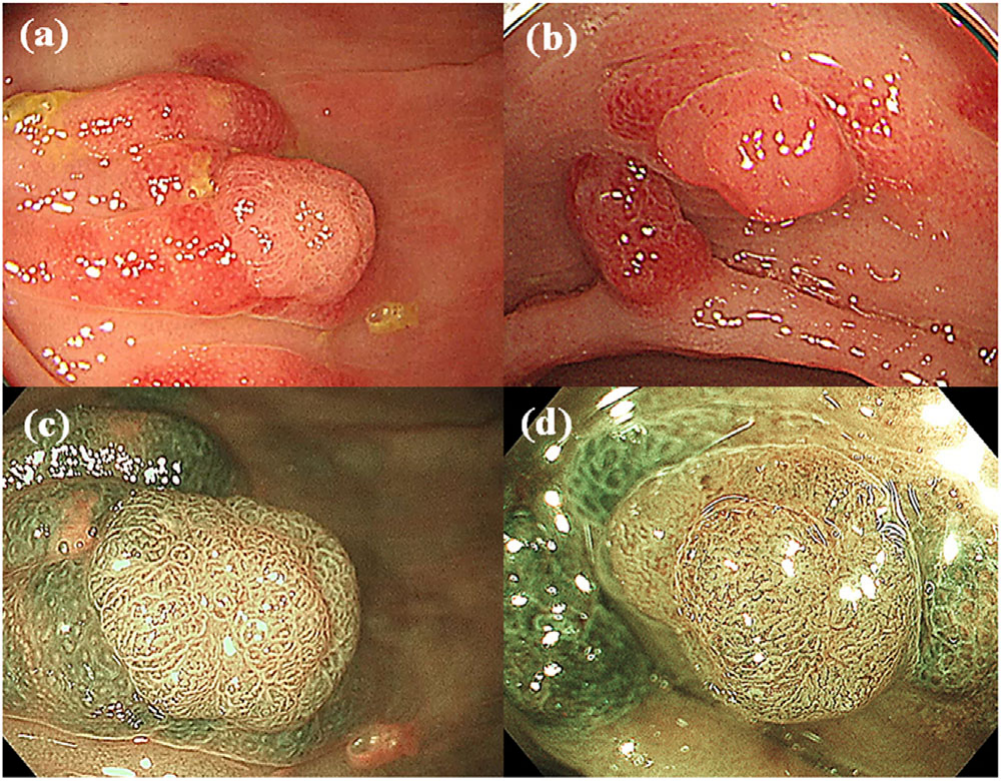
Colonoscopy showed adenoma coexisting with Cronkhite–Canada syndrome polyps (a, b). Magnified narrow‐band imaging revealed a regular distribution of microvessels and a regular reticular pattern on the adenoma; the microvessels of Cronkhite–Canada syndrome polyps developed a regularly elongated and network formation (c, d).

Esophagogastroduodenoscopy (EGD) revealed a dense strawberry‐like polyposis in the stomach, particularly across the lower gastric corpus to the antrum (Figure 1a). Total colonoscopy similarly revealed a dense strawberry‐like polyposis throughout the large intestine (Figure 1c). NBI magnification showed the sparse and dilated round pit on the polyps in both the stomach and large intestine (Figure 1b,d). The intervening mucosa was inflammatory and edematous. Histopathological analysis of biopsies from the stomach and colon polyps showed the edematous inflammatory interstitial tissue and expanded regular ductal structure, which was diagnosed as hamartomatous polyps. Based on these findings, the patient was diagnosed with CCS. There were countless CCS polyps, whereas adenomas without hamartoma components were not detected. However, twelve of the colorectal CCS polyps had a coexisting light reddish elevated component (Figure 2a,b). Magnifying NBI endoscopy revealed dilated round pits in the reddish area. On the other hand, the whitish components showed a regular distribution of microvessels and a regular reticular pattern satisfied the criteria for Type 2A of the Japan Narrow‐band‐imaging Expert Team classification, which is indicative of adenoma (Figure 2c,d). Endoscopic polypectomy was then performed on the 12 polyps.

**FIGURE 3:**
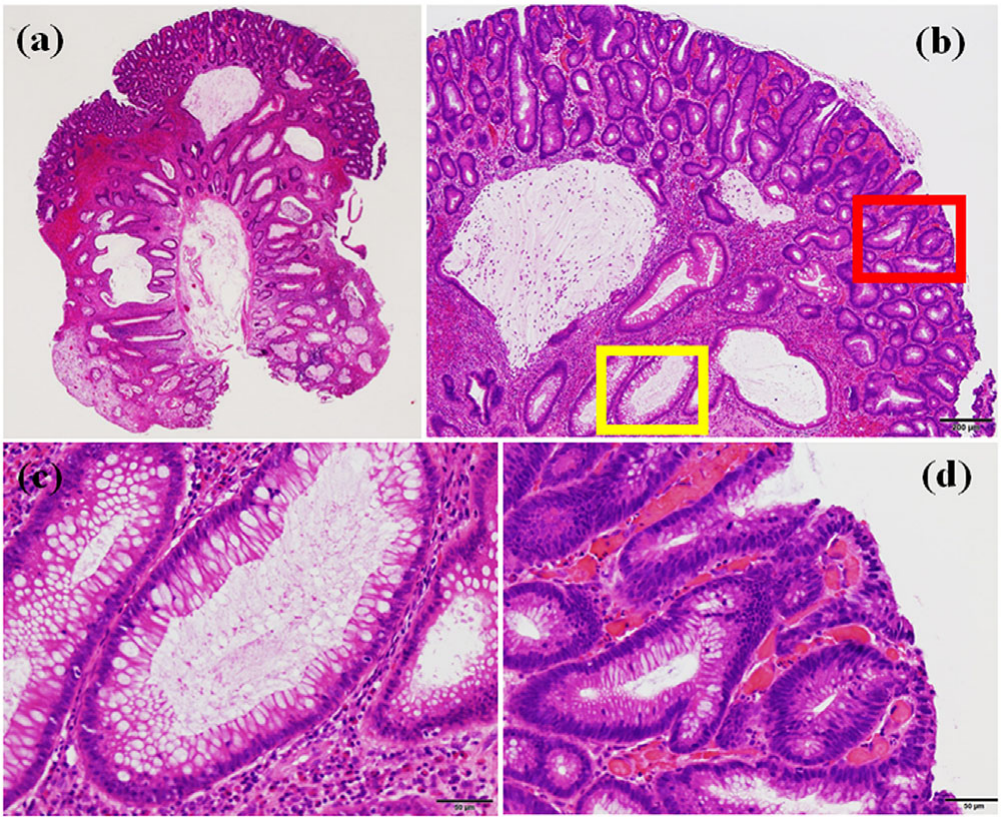
Histological findings. Polyps were a mixture of juvenile (hamartomatous) polyps and low‐grade adenoma (a, b). Cystically dilated crypts similar to juvenile (hamartomatous) polyps at the base of the polyp (b: yellow box, [c]). Dysplastic epithelium with hyperchromatic elongated nuclei compatible with low‐grade tubular adenoma (b: red box, [d]).

**FIGURE 4:**
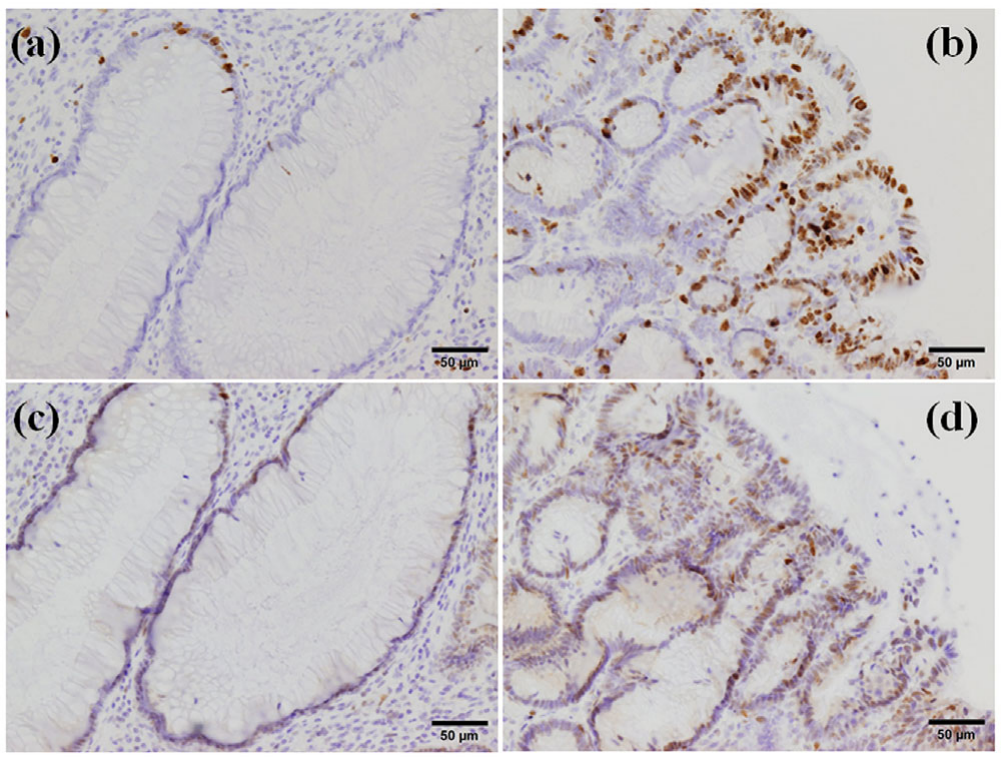
Pathological examination of the polyp. The paraffin section of polyps was stained with anti‐Ki‐67 antibody (a, b), and anti‐p53 antibody (c, d). Significantly higher labeling indices were seen in adenomatous lesions (b, d).

Pathological analysis showed that the polyps resected were all adenoma with hamartomatous components. In accordance with the NBI findings, while the base of the resected polyps consisted of hamartoma, the superficial layer contained a low‐grade tubular adenoma (Figure 3). Immunohistochemical analysis showed a significant increase in Ki‐67 index and p53 staining in adenomatous lesions, in contrast to the hamartomatous component (Figure 4). There was no difference in p53 staining between the areas bordering the adenoma and the more distant areas. The beta‐catenin staining pattern was similar in the hamartomatous and adenomatous lesions. The hamartomatous and adenomatous lesions were MUC5AC‐positive, but normal mucosa was negative (Figure S1a–c).

After the polypectomy, the patient underwent steroid therapies. Prednisolone was first administered from 30 mg/day, before gradual dose reduction and completion of treatment in ten months. Body weight and serum protein markers began to improve one month after medication began; this was concomitant with the amelioration of the taste disorder. The three‐month follow‐up esophagogastroduodenoscopy showed a reduction in the severity of strawberry‐like polyps, indicating a clear improvement in the gastric mucosal status. Six months after treatment began, a colonoscopy also revealed an improvement of the intestinal mucosa, showing only some small polyps observed as a hamartoma. Multiple hamartomatous polyps and CCS‐related symptoms have not reappeared in the 36 months since the steroid medication was terminated. In addition, obvious colorectal adenomas were not observed in periodic follow‐up endoscopies during the steroid treatment, suggesting that all adenomatous lesions were detected and resected before the medication.

## DISCUSSION

First reported by Cronkhite and Canada in 1955, CCS is mainly characterized by gastrointestinal hamartomatous polyposis, alopecia, hyperpigmentation, and onychodystrophy. Unlike several other common gastrointestinal polyposis syndromes, CCS is a non‐hereditary disease. The incidence rate is one in a million, with the most frequently affected populations being of Asian (and mainly Japanese) descent.^2^ The etiology remains still unknown, although infections, autoimmunity, vitamin deficiency, mental stress, and fatigue are all thought to play a part. Although the overall prognosis of CSS is poor with mortality rates as high as 55%, many cases respond to corticosteroid therapy.^3^, ^4^

The endoscopic and pathological assessment of gastrointestinal polyps plays a crucial role in the diagnosis process. Polyp pathology includes the following types: hyperplastic, adenomatous, hamartomatous (including juvenile), inflammatory, and serrated adenoma. The polyposis of CCS generally consists of hamartomatous polyps. In the present case, the gastrointestinal reddish polyps were pathologically determined as hamartoma, partially warranting the diagnosis of CCS. In addition, endoscopic findings showed that the intervening mucosa of this patient was inflammatory and edematous. This distribution pattern should be determined as a semi‐confluent type of CCS, according to the classification of CCS polyps by endoscopic findings.^5^

Although CCS is mainly comprised of hamartomatous polyps, the occasional coexistence and development of adenomas and carcinomas have been reported.^6^ Forty‐one percent of patients also have adenomas, which are precursor lesions to colorectal cancer; these patients have a 15% increased risk of cancer development.^7^ Since adenoma or early cancer commonly exists in the background of severe inflammation associated with CCS, it is difficult to detect such lesions endoscopically among the numerous hamartomatous polyps.^8^

Advanced endoscopic imaging techniques, such as NBI, magnifying endoscopy, and dye‐based contrast‐enhancement techniques, have been useful in detecting concurrent premalignant or malignant lesions. NBI is an endoscopic observation tool that captures the vascular structure of the mucosal surface layer, and it is useful in the diagnosis of adenoma or cancer.^9^ In the present case, we resected all of the 12 CCS polyps that were suspected as coexisting with adenoma by use of NBI magnification, which was histologically confirmed to harbor an adenoma component. We, therefore, consider that observation with magnified NBI endoscopy can facilitate the detection of adenomas or small cancers among the multiple CCS reddish polyps.

It has been speculated that adenomas develop in the epithelium adjacent to CCS‐specific polyps, which then progress to adenocarcinoma. There is no clear consensus on whether adenoma associated with CCS should be resected prior to steroid treatment.^5^ In this case, considering the oncogenic risk of the CCS‐associated adenoma during long‐term steroid treatment, we decided to resect the polyps prior to steroid administration.

There may be three possibilities for the coexistence of adenoma on CCS polyp. First, the adenoma develops from the epithelium of CCS polyps. Second, CCS polyps arise from adenomatous lesions. Third, the co‐occurrence is coincidental. Although it is beyond the scope of this case report to verify each possibility, Arima et al. reported a case of a colorectal Peutz‐Jeghers type polyp with hamartoma‐adenoma‐carcinoma sequence in a non‐Peutz‐Jeghers syndrome patient.^10^ A disease trajectory along the hamartoma‐adenoma‐carcinoma sequence has thus been suggested to explain the cancerous transformation in hamartomatous polyps. In addition, the rate of CCS‐related cancer was reported to be lower in patients with sustained endoscopic remission, compared to those with relapses or non‐responders.^5^ This suggests that chronic inflammation may induce colorectal neoplasia in CCS.

In conclusion, we could differentiate neoplastic lesions from multiple hamartomatous polyps prior to therapeutic intervention by using NBI magnified endoscopic observation. The use of NBI magnifying endoscopy may be useful in differentiating adenoma from CCS‐related polyps, allowing for early detection and treatment of precancerous lesions.

## CONFLICT OF INTEREST STATEMENT

None.

## Supporting information


**Figure S1**: MUC4AC expression in the CCS polyp. MUC5AC was positive in hamartomatous and adenomatous lesions (a,b), while it was negative in the normal mucosa (c).
