# Supplementary figures and images for: Narrow‐band imaging with magnification for the diagnosis of colorectal adenoma in a patient with Cronkhite‐Canada syndrome
Source: DEN Open. 2023 Jun 15;4(1):e257. doi: 10.1002/deo2.257 (PMC10272907; doi:10.1002/deo2.257)

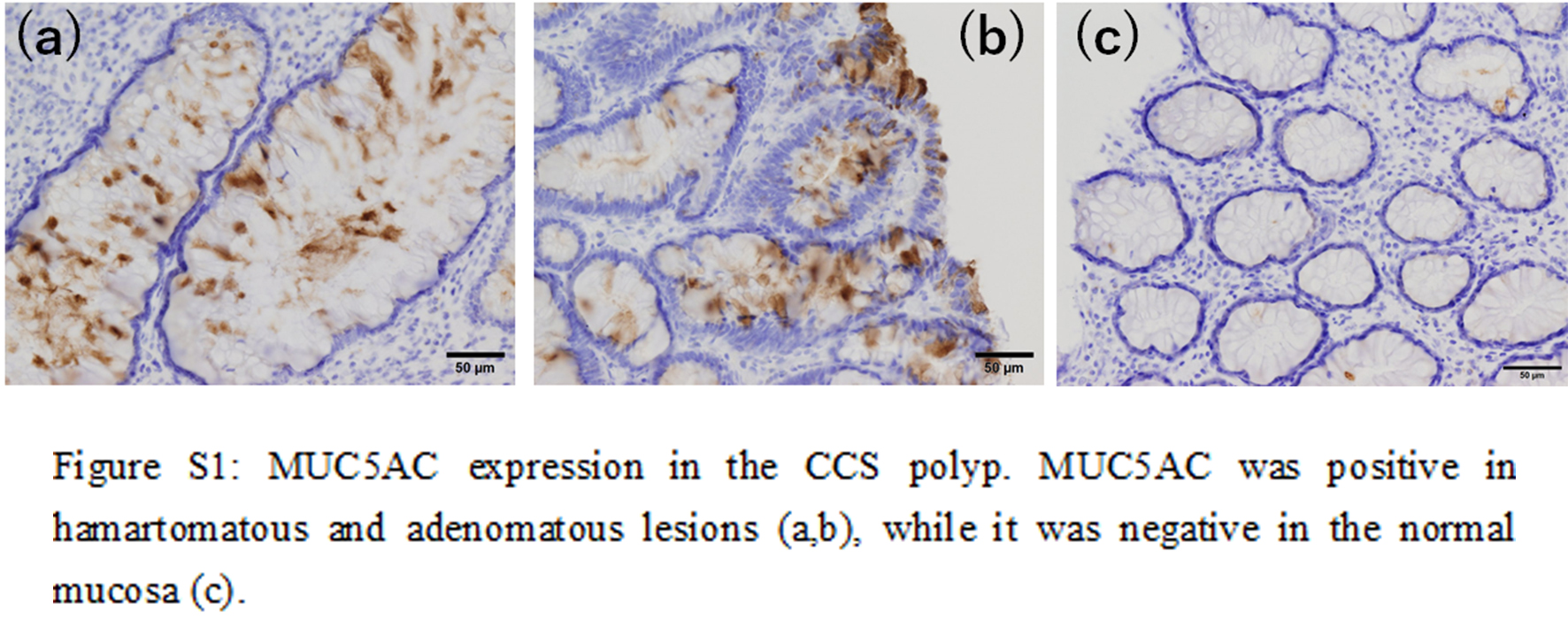

Supplement: Supplementary file 1 — Figure S1: MUC4AC expression in the CCS polyp. MUC5AC was positive in hamartomatous and adenomatous lesions (a,b), while it was negative in the normal mucosa (c). [file DEO2-4-e257-s001.jpg]
